# Deep learning for neuroimaging: a validation study

**DOI:** 10.3389/fnins.2014.00229

**Published:** 2014-08-20

**Authors:** Sergey M. Plis, Devon R. Hjelm, Ruslan Salakhutdinov, Elena A. Allen, Henry J. Bockholt, Jeffrey D. Long, Hans J. Johnson, Jane S. Paulsen, Jessica A. Turner, Vince D. Calhoun

**Affiliations:** ^1^The Mind Research NetworkAlbuquerque, NM, USA; ^2^Department of Computer Science, University of New MexicoAlbuquerque, NM, USA; ^3^Department of Computer Science, University of TorontoToronto, ON, Canada; ^4^Department of Biological and Medical Psychology, University of BergenBergen, Norway; ^5^Advanced Biomedical Informatics Group, LLC, University of IowaIowa City, IA, USA; ^6^Department of Psychiatry, Carver College of Medicine, University of IowaIowa City, IA, USA; ^7^Department of Biostatistics, College of Public Health, University of IowaIowa City, IA, USA; ^8^Department of Biomedical Engineering, College of Engineering, University of IowaIowa City, IA, USA; ^9^Department of Psychology, Neuroscience Institute, University of IowaIowa City, IA, USA; ^10^Department of Neurology, Carver College of Medicine, University of IowaIowa City, IA, USA; ^11^Department of Psychology, Neuroscience Institute, Georgia State UniversityAtlanta, GA, USA; ^12^Department of Electrical and Computer Engineering, University of New MexicoAlbuquerque, NM, USA

**Keywords:** MRI, fMRI, intrinsic networks, classification, unsupervised learning

## Abstract

Deep learning methods have recently made notable advances in the tasks of classification and representation learning. These tasks are important for brain imaging and neuroscience discovery, making the methods attractive for porting to a neuroimager's toolbox. Success of these methods is, in part, explained by the flexibility of deep learning models. However, this flexibility makes the process of porting to new areas a difficult parameter optimization problem. In this work we demonstrate our results (and feasible parameter ranges) in application of deep learning methods to structural and functional brain imaging data. These methods include deep belief networks and their building block the restricted Boltzmann machine. We also describe a novel constraint-based approach to visualizing high dimensional data. We use it to analyze the effect of parameter choices on data transformations. Our results show that deep learning methods are able to learn physiologically important representations and detect latent relations in neuroimaging data.

## 1. Introduction

One of the main goals of brain imaging and neuroscience—and, possibly, of most natural sciences—is to improve understanding of the investigated system based on data. In our case, this amounts to inference of descriptive features of brain structure and function from non-invasive measurements. Brain imaging field has come a long way from anatomical maps and atlases toward data driven feature learning methods, such as seed-based correlation (Biswal et al., 1995), canonical correlation analysis (Sui et al., 2012), and independent component analysis (ICA) (Bell and Sejnowski, 1995; McKeown et al., 1998). These methods are highly successful in revealing known brain features with new details (Brookes et al., 2011) (supporting their credibility), in recovering features that differentiate patients and controls (Potluru and Calhoun, 2008) (assisting diagnosis and disease understanding), and starting a “resting state” revolution after revealing consistent patterns in data from uncontrolled resting experiments (Raichle et al., 2001; van den Heuvel and Hulshoff, 2010). Classification of human brain data is typically used merely as a way to evaluate the performance of a proposed feature (e.g., percent signal change of an activation map within a set of ROIs, identification of a subset of voxels, or a specific network of interest such as default mode) relative to previously proposed features. Features (and feature selection approaches) are used since classification methods—including the most accurate ones—do not often perform well on raw data and, when they do, the reasons for their accuracy are rarely intuitive or informative. Commonly, if that answer's accuracy improves when a new discriminative feature (biomarker) is proposed, this biomarker is considered an improvement. While a perfect classification approach would be of use, the process of suggesting biomarker candidates would still be a subjective and difficult process.

Typical approaches to classification (including the current multi-voxel classification approaches which are popular in brain imaging) must be preceded by a feature selection step which is not needed for deep learning methods. Deep learning methods are breaking records in the areas of speech, signal, image, video and text mining and recognition and improving state of the art classification accuracy by, sometimes, more than 30% where the prior decade struggled to obtain a 1–2% improvements (Krizhevsky et al., 2012; Le et al., 2012). What differentiates them from other classifiers, however, is the automatic feature learning from data which largely contributes to improvements in accuracy. This represents an important advantage and removes a level of subjectivity (e.g., the researcher typically has to decide which features should be tried) from existing approaches. With deep learning this subjective step is avoided.

Another distinguishing feature of deep learning is the depth of the models. Based on already acceptable feature learning results obtained by shallow models—currently dominating neuroimaging field—it is not immediately clear what benefits would depth have. Considering the state of multimodal learning, where models are either assumed to be the same for analyzed modalities (Moosmann et al., 2008) or cross-modal relations are sought at the (shallow) level of mixture coefficients (Liu and Calhoun, 2007), deeper models better fit the intuitive notion of cross-modality relations, as, for example, relations between genetics and phenotypes should be indirect, happening at a deeper conceptual level.

In this work we present our recent advances in application of deep learning methods to functional and structural magnetic resonance imaging (fMRI and sMRI). Each consists of brain volumes but for sMRI these are static volumes—one per subject/session,—while for fMRI a single subject dataset is comprised of multiple volumes capturing the changes during an experimental session. Our goal is to validate feasibility of this application by (a) investigating if a building block of deep generative models—a restricted Boltzmann machine (RBM) (Hinton, 2000)—is competitive with ICA (a representative model of its class) (Section 2); (b) examining the effect of the depth in deep learning analysis of structural MRI data (Section 3.3); and (c) determining the value of the methods for discovery of latent structure of a large-scale (by neuroimaging standards) dataset (Section 3.4). The measure of feature learning performance in a shallow model (a) is comparable with existing methods and known brain physiology. However, this measure cannot be used when deeper models are investigated. As we further demonstrate, classification accuracy does not provide the complete picture either. To be able to visualize the effect of depth and gain an insight into the learning process, we introduce a flexible constraint satisfaction embedding method that allows us to control the complexity of the constraints (Section 3.2). Deliberately choosing local constraints we are able to reflect the transformations that the deep belief network (DBN) (Hinton and Salakhutdinov, 2006) learns and applies to the data and gain additional insight.

## 2. A shallow belief network for feature learning

**Figure 1:**
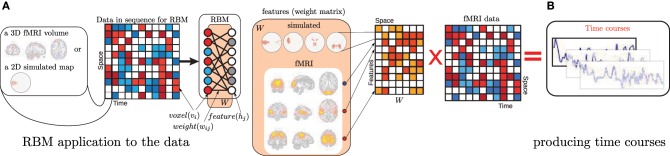
**(A)** An illustration of how an RBM is applied to the data as well as a graphical representation of RBM's structure. **(B)** Demonstrates the way we produce time courses from the data and learned features, which is simply a projection of data into the feature space. Note, for time course computation in Section 2 we do not apply the hidden units' non-linearity after this projection, while in Section 3 the complete feed-forward processing is realized.

Prior to investigating the benefits of depth of a DBN in learning representations from fMRI and sMRI data, we would like to find out if a shallow (single hidden layer) model–which is the RBM—from this family meets the field's expectations. As mentioned in the introduction, a number of methods are used for feature learning from neuroimaging data: most of them belong to the single matrix factorization (SMF) class. We do a quick comparison to a small subset of SMF methods on simulated data; and continue with a more extensive comparison against ICA as an approach trusted in the neuroimaging field. Similarly to RBM, ICA relies on the bipartite graph structure, or even is an artificial neural network with sigmoid hidden units as is in the case of Infomax ICA (Bell and Sejnowski, 1995) that we compare against. Note the difference with RBM: ICA applies its weight matrix to the (shorter) temporal dimension of the data imposing independence on the spatial dimension while RBM applies its weight matrix (hidden units “receptive fields”) to the high dimensional spatial dimension instead (Figure 1). Each row of the weight matrix of an RBM [as expressed in (1)] is a receptive field of a hidden unit: it has the dimensions of space (volume) and the magnitude of the values indicates regions the unit is tuned to (when trained). These weights are our features uniquely assigned to a hidden unit. Reflecting this we interchangeably call the rows of *W* and corresponding hidden units “features” always meaning “receptive fields.”

### 2.1. A restricted boltzmann machine

A *restricted Boltzmann machine* (RBM) is a Markov random field that models data distribution parameterizing it with the Gibbs distribution over a bipartite graph between visible *v* and hidden variables *h* (Fischer and Igel, 2012): *p*(*v*) = ∑_*h*_ *p*(*v*, *h*) = ∑_*h*_ 1/*Z* exp(− *E*(*v*, *h*)), where *Z* = ∑_*v*_ ∑_*h*_ *e*^−*E*(*v*, *h*^) is the normalization term (the partition function) and *E*(*v*, *h*) is the energy of the system. Each visible variable in the case of fMRI data represents a voxel of an fMRI scan with a real-valued and approximately Gaussian distribution. In this case, the energy is defined as:

(1) $$E(v,h)\;=-\sum_{ij}\;\frac{v_{j}}{\sigma_{j}}\;W_{ji}h_{i}\;-\;\sum_{j}\;\frac{{\left(a_{j}-v_{j}\right)}^{2}}{\sigma_{j}^{2}}\;-\;\sum_{i}\;b_{i}h_{i},$$

where *a*_*j*_ and *b*_*i*_ are biases and σ_*j*_ is the standard deviation of a parabolic containment function for each visible variable *v*_*j*_ centered on the bias *a*_*j*_. In general, the parameters σ_*j*_ need to be learned along with the other parameters. However, in practice normalizing the distribution of each voxel to have zero mean and unit variance is faster and yet effective (Nair and Hinton, 2010). A number of choices affect the quality of interpretation of the representations learned from fMRI by an RBM. Encouraging sparse features via the *L*_1_-regularization: λ‖*W*‖_1_ (λ = 0.1 gave best results) and using hyperbolic tangent for hidden units non-linearity are essential settings that respectively facilitate spatial and temporal interpretation of the result.

*L*_1_ regularization is a useful tool in automated feature learning as it can reduce overfitting. It adds an additional gradient term which forces most of the weights to be zero while allowing a few of the weights to grow large (Hastie et al., 2009). The update rule at a data point ***x***_*n*_ becomes:

(2) $$W_{ij}\to W_{ij}+\;\epsilon \;(\frac{\partial }{\partial W_{ij}}\;\log\;p(v=x_{n})-\lambda \;\cdot \;\mathrm{sgn}\;(W_{ij})),$$

where ϵ is the learning rate.

In the case of fMRI, spatial features have similar interpretation whether their activity is below or above the baseline at a given time. However, the more common logistic hidden units are unable to adequately represent a feature with activity that crosses the baseline boundary. To model these features with non-negative hidden units, RBM divides the work among two units, one with a positive and another with—often slightly different due to differences in the exhibitory and excitatory behaviors—negative receptive fields. This is not entirely desirable, as splitting intrinsic spatial networks along a distribution mean hinders the interpretive power of the model. The *hyperbolic tangent* is an alternative function in the exponential family, which has some similar properties to the logistic function when used to model the conditional probabilities of hidden units. However, a key difference is the output is sampled from {−1, 1}. Sign of the receptive fields then is completely symmetric with respect to hidden variable sign: positive receptive fields will generate with positive hidden variables, while negative receptive fields will generate with negative receptive fields. An additional consequence of this is that a single hidden unit can generate samples over the normal distribution solving the problem of learning duplicate features of opposite signs.

To estimate parameters ***W***, *a*, and *b* we need to maximize their log likelihood. In the case of RBM the gradient of the log likelihood with respect to the parameters has a closed form. However, it involves an intractable expectation over the joint distribution of visible and hidden units that appear because of the partition function *Z*. To deal with this an approximation to the gradient is usually employed. We use the truncated Gibbs sampling method called contrastive divergence (CD) with a single sampling step (CD-1). Further information on RBM model can be found in Hinton (2000); Hinton et al. (2006).

Note, that all of the parameter choices and modifications to the original RBM algorithm (e.g., the regularization) employed in this work are already conveniently implemented in a freely available package *deepnet*: https://github.com/nitishsrivastava/deepnet. All parameters can be set as part of the model specification. We believe, for neuroimaging research it is more productive to use this (or other available) package rather than engaging into an implementation.

The way RBM is applied to the data is consistent across this paper: visible units “see” voxels. Figure 1 illustrates the process of RBM application to the data (in training and in the feed-forward mode of Section 3) and clarifies what we treat as features both for simulated and fMRI datasets. Although in Section 3 we use structural data and time dimension of the figure is the subjects, the manner of RBM application (in pre-training) is identical. Note, expression (1) addresses visible and hidden units with different subscript (*j* and *i* respectively). For each hidden unit *i* and visible unit *j* there is a weight parameter *W*_*ij*_. There are as many visible units as there are voxels and each hidden unit has as many weight values. These weights are our features. They are sometimes called receptive fields or filters.

### 2.2. Synthetic data

In this section we summarize our comparisons of RBM with SMF models—including Infomax ICA (Bell and Sejnowski, 1995), PCA (Hastie et al., 2009), sparse PCA (sPCA) (Zou et al., 2006), and sparse NMF (sNMF) (Hoyer, 2002)—on synthetic data with known spatial maps generated to simulate fMRI. The SimTB toolbox (Erhardt et al., 2012) was used to generate synthetic 3D (x, y, and t) fMRI-like data from linear combinations of 27 distinct spatial sources with 2D Gaussian spatial profiles. Rician noise was added to the combined data to achieve a contrast-to-noise ratio between 0.65 and 1. Data for 20 artificial “subjects" consisting of 128 volumes were generated from the auditory oddball (AOD) example experiment from the toolbox, in which a subset of sources are modulated by “standard,” “target,” and “novel” events with different weights. Additionally, two sources are modulated by nearly identical noise (spike) events. Thus, source activations are temporally correlated to some degree, though each has its own unique behavior.

RBMs were constructed with 16936 Gaussian visible units (one for each voxel in the 128 × 128 image), and a variable number of hyperbolic tangent hidden units. The *L*_1_ decay rate λ was set to 0.1 based on performance over multiple experiments, and the learning rate ϵ was set to 0.08. The RBMs were then trained with a batch size of 5 for approximately 75 epochs to allow for full convergence of the parameters.

**Figure 2:**
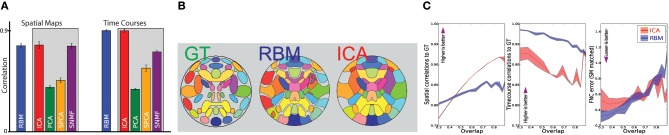
**Comparison of RBM estimation accuracy of features and their time courses with SMFs. (A)** Average spatial map (SM) and time course (TC) correlations to ground truth for RBM and SMF models (gray box). **(B)** Ground truth (GT) SMs and estimates obtained by RBM and ICA (thresholded at 0.4 height). Colors are consistent across the methods. Gray indicates background or areas without SMs above threshold. **(C)** Spatial, temporal, and cross correlation (FNC) accuracy for ICA (red) and RBM (blue), as a function of spatial overlap of the true sources from **(B)**. Lines indicate the average correlation to GT, and the color-fill indicates ±2 standard errors around the mean.

We found that for simulated data, RBM captures the features better with the number of hidden units higher that the true model order. We use terms “model rank” and “model order” interchangeably to mean the number of hidden units in RBM or number of independent components for ICA. For this dataset RBM's performance in spatial map estimation has stabilized near model order 60. We set the model order to 64 as the GPU implementation of RBM favors model orders of powers of 2. After investigating performance of ICA (as well as sNMF and PCA) under various model orders we did not observe a performance decrease for these approaches (with respect to their best performing model order) at the value of 64. Figure 2 presents result where the model order for all models was set to this value.

Figure 2A shows the correlation of spatial maps (SM) and time course (TC) estimates to the ground truth for RBM, ICA, PCA, sPCA, and sNMF. Allowing model orders to differ from the ground truth, features were matched to the ground truth by solving the assignment problem using the Hungarian algorithm (West, 2001) based on maximizing absolute positive correlation of SMs to the ground truth. Correlations are averaged across all sources and datasets. RBM and ICA showed the best overall performance. While sNMF also estimated SMs well, it showed inferior performance on TC estimation, likely due to the non-negativity constraint. Based on these results and the broad adoption of ICA in the field, we focus on comparing Infomax ICA and RBM.

Figure 2B shows the full set of ground truth sources along with RBM and ICA estimates for a single representative dataset. SMs are thresholded and represented as contours for visualization.

For Figure 2C twelve sets of SimTB data were produced by varying a SimTB source “spread” parameter, which changes the relative spatial standard deviation of each source. Increase in spread increases the percentage of *overlap* between features; we define the total overlap of a set of sources as the percentage of voxels in which more than one source contributes over 0.5 standard deviations. We constructed datasets with overlap ranging from 0.3 (minimal spatial overlap between sources) and 0.88 (very high overlap). Results showed similar performance for RBM and ICA (Figure 2C), with a slight advantage for ICA with regard to SM estimation, and a slight advantage for RBM with regards to TC estimation. RBM and ICA also showed comparable performance estimating cross correlations also called functional network connectivity (FNC). FNC is a measure of interaction between intrinsic networks of the brain (Allen et al., 2012). In our case this amounts to cross-correlations of subject specific time courses of each of the hidden unit expressed in a correlation matrix.

### 2.3. An fMRI data application

Data used in this work comprised of task-related scans from 28 (five females) healthy participants, all of whom gave written, informed, IRB-approved consent at Hartford Hospital and were compensated for participation^1^. All participants were scanned during an auditory oddball task (AOD) involving the detection of an infrequent target sound within a series of standard and novel sounds^2^.

Scans were acquired at the Olin Neuropsychiatry Research Center at the Institute of Living/Hartford Hospital on a Siemens Allegra 3T dedicated head scanner equipped with 40mTm gradients and a standard quadrature head coil (Calhoun et al., 2008; Swanson et al., 2011). The AOD consisted of two 8-min runs, and 249 scans (volumes) at 2 s TR (0.5 Hz sampling rate) were used for the final dataset. Data were post-processed using the SPM5 software package (Friston et al., 1994), motion corrected using INRIalign (Freire et al., 2002), and subsampled to 53 × 63 × 46 voxels. The complete fMRI dataset was masked below mean and the mean image across the dataset was removed, giving a complete dataset of size 70969 voxels by 6972 volumes. Each voxel was then normalized to have zero mean and unit variance.

The RBM was constructed using 70969 Gaussian visible units and 64 hyperbolic tangent hidden units. The hyper parameters ϵ (0.08 from the searched [1 × 10^−4^, 1 × 10^−1^] range) for learning rate and λ (0.1 from the searched range [1 × 10^−4^, 1 × 10^−1^]) for *L*_1_ weight decay were selected as those that showed a reduction of reconstruction error over training and a significant reduction in span of the receptive fields respectively. Parameter value outside the ranges either resulted in unstable or slow learning (ϵ) or uninterpretable features (λ). The RBM was then trained with a batch size of 5 for approximately 100 epochs to allow for full convergence of the parameters.

After flipping the sign of negative receptive fields, we then identified and labeled spatially distinct features as corresponding to brain regions with the aid of AFNI (Cox, 1996) excluding features which had a high probability of corresponding to white matter, ventricles, or artifacts (e.g., motion, edges). Note, the sign flipping is strictly parallel to what is done to ICA results in order to address the “sign ambiguity,” where both signs of the spatial map and the time course are flipped. In the RBM case, only spatial maps are flipped explicitly (i.e., multiplied by −1) but the time courses get the correct sign automatically.

We normalized the fMRI volume time series to mean zero and used the trained RBM in feed-forward mode to compute time series for each fMRI feature. This was done to better compare to ICA, where the mean is removed in PCA preprocessing.

**Figure 3:**
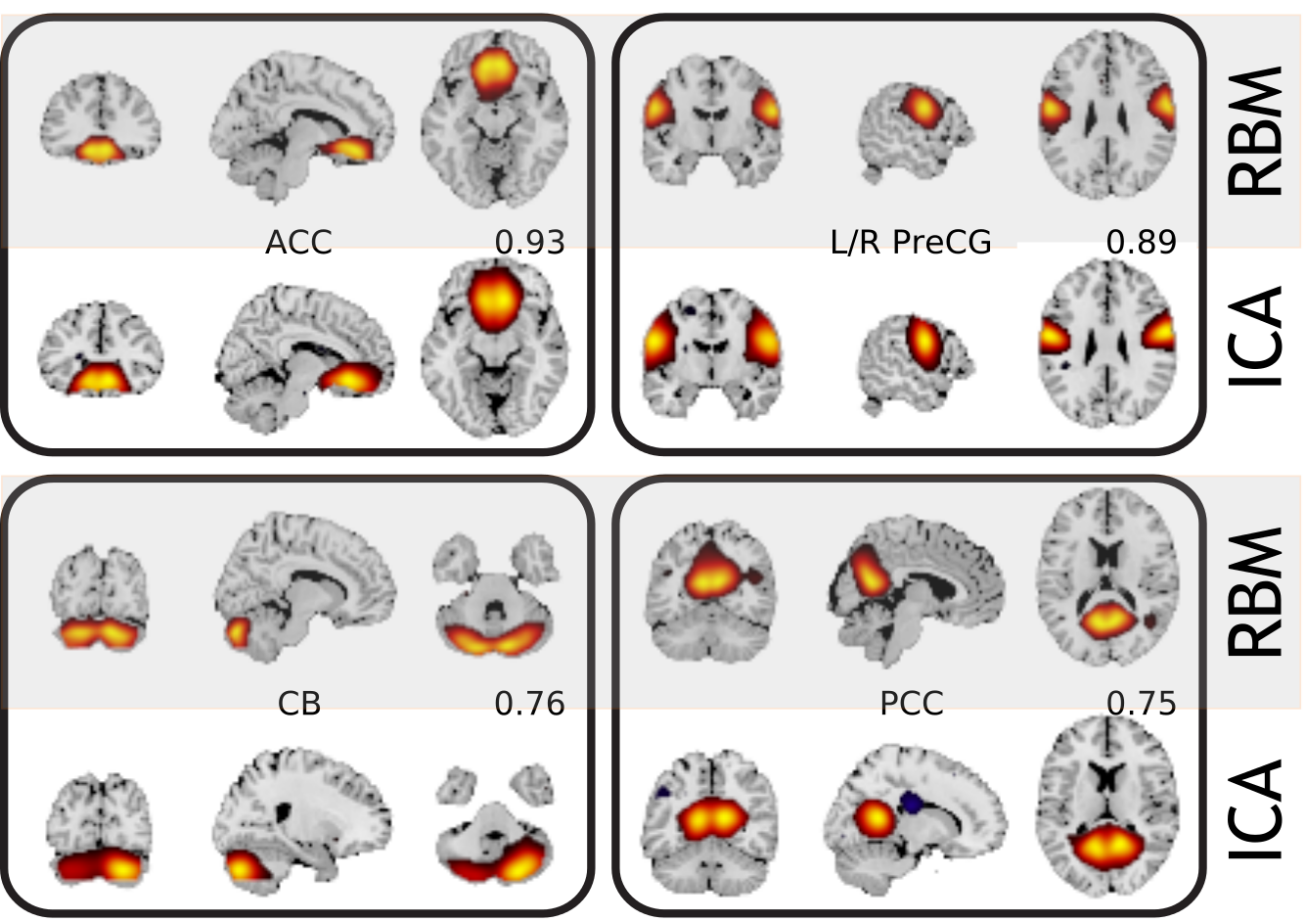
**Intrinsic brain networks estimated by ICA and RBM**.

The work-flow is outlined in Figure 1, while Figure 3 shows comparison of resulting features with those obtained by Infomax ICA. In general, RBM performs competitively with ICA, while providing–perhaps, not surprisingly due to the used *L*_1_ regularization—sharper and more localized features. While we recognize that this is a subjective measure we list more features in Figure S2 of Section 5 and note that RBM features lack negative parts for corresponding features. Note, that in the case of *L*_1_ regularized weights RBM algorithms starts to resemble some of the ICA approaches (such as the recent RICA by Le et al., 2011), which may explain the similar performance. However, the differences and possible advantages are the generative nature of the RBM and no enforcement of component orthogonality (not explicit at the least). Moreover, the block structure of the correlation matrix (see below the Supplementary material section) of feature time courses provide a grouping that is more physiologically supported than that provided by ICA. For example, see Figure S1 in the Supplementary Material section below. Perhaps, because ICA working hard to enforce spatial independence subtly affects the time courses and their cross-correlations in turn. We have observed comparable running times of the (non GPU) ICA (http://www.nitrc.org/projects/gift) and a GPU implementation of the RBM (https://github.com/nitishsrivastava/deepnet). This, however, is not an exhaustive comparison as there are other important metrics such as stability of the learned features (Zuo et al., 2010) that may better differentiate RBM from the popular models. Some of alternative comparison metrics for evaluating RBM against the state of the art were considered by Hjelm et al. (2014).

## 3. Validating the depth effect

Since the RBM results demonstrate a feature-learning performance competitive with the state of the art (or better), we proceed to investigating the effects of the model depth. To do that we turn from fMRI to sMRI data. As it is commonly assumed in the deep learning literature (Le Roux and Bengio, 2010) the depth is often improving classification accuracy. We investigate if that is indeed true in the sMRI case. Structural data is convenient for the purpose as each subject/session is represented only by a single volume that has a label: control or patient in our case. Compare to 4D data where hundreds of volumes belong to the same subject with the same disease state.

### 3.1. A deep belief network

A DBN is a sigmoidal belief network (although other activation functions may be used) with an RBM as the top level prior. The joint probability distribution of its visible and hidden units is parametrized as follows:

(3) $$\begin{array}{l} P\;(v,\;h^{1},\;h^{2},\ldots ,\;h^{l})=P\;(v|h^{1})\;P(h^{1}|h^{2})\cdots P\;(h^{l\;-\;2},\;h^{l\;-\;1})\\ \;P\;(h^{l\;-\;1},\;h^{l}), \end{array}$$

where *l* is the number of hidden layers, *P*(*h*^*l* − 1^, *h*^*l*^) is an RBM, and *P*(*h*^*i*^|*h*^*i* + 1^) factor into individual conditionals:

(4) $$P\;(h^{i}|h^{i+1})=\prod_{j=1}^{n_{i}}P\;(h_{j}^{i}|h^{i+1})$$

The important property of DBN for our goals of feature learning to facilitate discovery is its ability to operate in generative mode with fixed values on chosen hidden units thus allowing one to investigate the features that the model have learned and/or weighs as important in discriminative decisions. We, however, are not going to use this property in this section, focusing instead on validating the claim that a network's depth provides benefits for neuroimaging data analysis. And we will do this using discriminative mode of DBN's operation as it provides an objective measure of the depth effect.

**Figure 4:**
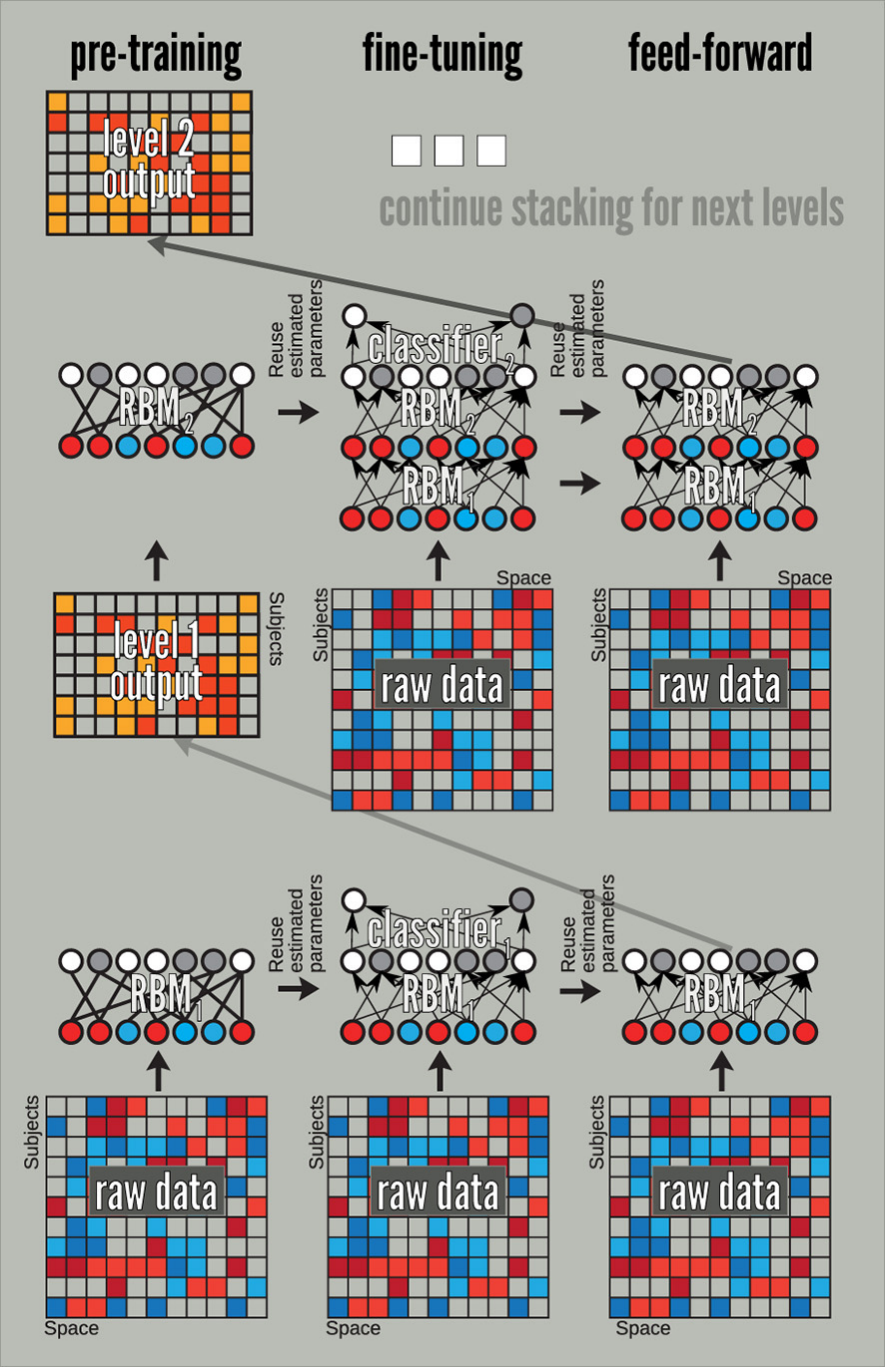
**A pre-train/fine-tune sequence for training a DBN**. The pre-training stage uses the data from the previous layer, which for our 3-level DBN amounts to raw data (for layer 1), and outputs of the feed-forward mode of the fine-tuned RBMs. The fine-tuning and feed-forward stages always start from the raw data. The fine-tuning stage initializes RBM weights with those learned at the pre-training.

DBN training splits into two stages: pre-training and discriminative fine tuning. A DBN can be pre-trained by treating each of its layers as an RBM—trained in an unsupervised way on inputs from the previous layer—and later fine-tuned by treating it as a feed-forward neural network. The latter allows supervised training via the error back propagation algorithm. We use this schema in the following by augmenting each DBN with a soft-max layer at the fine-tuning stage. The overall approach is outlined in Figure 4. Although, we only show there how we construct and train DBN's of depth 1 and 2, the process can be continued and DBNs of larger depth can be built. We do so when we build the third layer of the DBN employed in the experiments of this section.

While fMRI data of Section 2.3 was not too similar to the natural images—the traditional domain of DBN application—the structural MRI resembles the images to a large extent. In particular, in this section we use gray matter concentration maps: a point in this map (a voxel) contains intensity values much like in a monochrome image. This similarity allows us to import some of the parameters traditionally used in image processing DBNs: logistic hidden unit non-linearity and the dropout trick (Hinton et al., 2012).

### 3.2. Non-linear embedding as a constraint satisfaction problem

A DBN and an RBM operate on data samples, which are brain volumes in the fMRI and sMRI case. A 5 min fMRI experiment with 2 s sampling rate yields 150 of these volumes per subject. For sMRI studies number of participating subjects varies but in this paper we operate with a 300 and a 3500 subject-volumes datasets. Transformations learned by deep learning methods do not look intuitive in the hidden node space and generative sampling of the trained model does not provide a sense if a model have learned anything useful in the case of MRI data: in contrast to natural images, fMRI and sMRI images do not look very intuitive. Instead, we use a non-linear embedding method to control whether a model learned useful information and to assist in investigation of what have it, in fact, learned.

One of the purposes of an embedding is to display a complex high dimensional dataset in a way that is (i) intuitive, and (ii) representative of the data sample. The first requirement usually leads to displaying data samples as points in a 2-dimensional map, while the second is more elusive and each approach addresses it differently. Embedding approaches include relatively simple random linear projections—provably preserving some neighbor relations (de Vries et al., 2010)—and a more complex class of non-linear embedding approaches (Sammon, 1969; Roweis and Saul, 2000; Tenenbaum et al., 2000; Van der Maaten and Hinton, 2008). In an attempt to organize the properties of this diverse family we have aimed at representing non-linear embedding methods under a single constraint satisfaction problem (CSP) framework (see below). We hypothesize that each method places the samples in a map to satisfy a specific set of constraints. Although this work is not yet complete, it proven useful in our current study. We briefly outline the ideas in this section to provide enough intuition of the method that we further use in Section 3.

Since we can control the constraints in the CSP framework, to study the effect of deep learning we choose them to do the least amount of work—while still being useful—letting the DBN do (or not) the hard part. A more complicated method such as t-SNE (Van der Maaten and Hinton, 2008) already does complex processing to preserve the structure of a dataset in a 2D map – it is hard to infer if the quality of the map is determined by a deep learning method or the embedding. While some of the existing method may have provided the “least amount of work” solutions as well we chose to go with the CSP framework. It explicitly states the constraints that are being satisfied and thus lets us reason about deep learning effects within the constraints, while with other methods—where the constraints are implicit—this would have been harder.

A constraint satisfaction problem (CSP) is one requiring a solution that satisfies a set of constraints. One of the well known examples is the boolean satisfiability problem (SAT). There are multiple other important CSPs such as the packing, molecular conformations, and, recently, error correcting codes (Derbinsky et al., 2013). Freedom to setup per-point constraints without controlling for their global interactions makes a CSP formulation an attractive representation of the non-linear embedding problem. Pursuing this property we use the iterative “divide and concur” (DC) algorithm (Gravel and Elser, 2008) as the solver for our representation. In DC algorithm we treat each point on the solution map as a variable and assign a set of constraints that this variable needs to satisfy (more on these later). Then each points gets a “replica” for each constraint it is involved into. In our case, this means that for *n* points each point will have *n* replicas as we have a constraint per point. Then DC algorithm alternates the divide and concur projections. The divide projection moves each “replica” points to the nearest locations in the 2D map that satisfy the constraint they participate in. More specifically, *k*-neighbors of the point in the *d*-dimensional space are moved in the direction of the point in the 2D map by a step proportional to their distance to this point in the data space (in our case, this is DBN representation space). This is a soft constraint as opposed to just forcing the *k*-neighbors to be the nearest neighbors in the 2D map. The concur projection concurs locations of all “replicas” of a point by placing them at the average location on the map. The key idea is to avoid local traps by combining the divide and concur steps within the difference map (Elser et al., 2007). A single location update is represented by:

(5) $$\begin{array}{l} x_{c}=P_{c}\;((1+1/\beta )\;*\;P_{d}(x)\;-\;1/\beta \;*\;x)\\ x_{d}=P_{d}\;((1-1/\beta )\;*\;P_{c}\;(x)\;+\;1/\beta \;*\;x)\\ x\;=x\;+\;\beta \;*\;(x_{c}-x_{d}), \end{array}$$

where *P*_*d*_(·) and *P*_*c*_(·) denote the divide and concur projections and β is a user-defined parameter.

The concur projection *P*_*c*_(·) that we use throughout the paper simply averages locations of all replicas of the point in the 2D map and assigns all of the replicas to this new location. While the concur projection will only differ by subsets of “replicas” across different methods representable in DC framework, the divide projection *P*_*d*_(·) is unique and defines the algorithm behavior. In this paper, we choose a divide projection that keeps *k* nearest neighbors of each point in the higher dimensional space also its neighbors in the 2D map. This is a simple local neighborhood constraint that allows us to assess effects of deep learning transformation leaving most of the mapping decisions to the deep learning. Each point in the 2D map is pulling it's *k*-neighbors into its neighborhood until an equilibrium is reached and the points stop regrouping (much). With this we strive for the best nearest neighbor representation of the *d*-dimensional map in the 2D space. The changes to the nearest neighborhood are performed by the DBN, since our algorithm does not affect this information.

We found that hard constraints often do not lead to a solution getting on a widely oscillating path from initial iterations. We have observed this while investigating (a)placing all of the neighbors of a replica in 2D at the same distance as they are in the source space and (b)ensuring that k-neighbors of a replica are the same as in the source space. The approach that converges to most interpretable results (with respect to the non-neuroimaging data we tuned it on) simply pulls the source space neighbors at each iteration toward the replica in 2D. Choice of *k*, as we have found, does not determine separation of data clusters as much as their shape. Smaller values of *k* lead to more elongated groups which turn into rays in the extreme case, while larger *k* leads to more spherical maps. In **Figure 6** and in **Figure 8** we use *k* = 160, which for the larger dataset of Section 3.4 leads to more elongated groups.

Note, that for a general dataset we may not be able to satisfy this constraint: each point has exactly the same neighbors in 2D as in the original space (and this is what we indeed observe). The DC algorithm, however, is only guaranteed to find the solution if it exists and oscillates otherwise. Oscillating behavior is interesting, as it is detectable and could be used to stop the algorithm. Letting the algorithm run while observing real time changes to the 2D map may provide additional information about the structure of the data. Another practically important feature of the algorithm: it is deterministic. Given the same parameters [β and the parameters of *P*_*d*_(·)] it converges to the same solution regardless of the initial point. If each of the points participates in each constraint then complexity of the algorithm is quadratic. With our simple *k* neighborhood constraints it is *O*(*kn*), for *n* samples/points.

### 3.3. A schizophrenia structural MRI dataset

**Figure 5:**
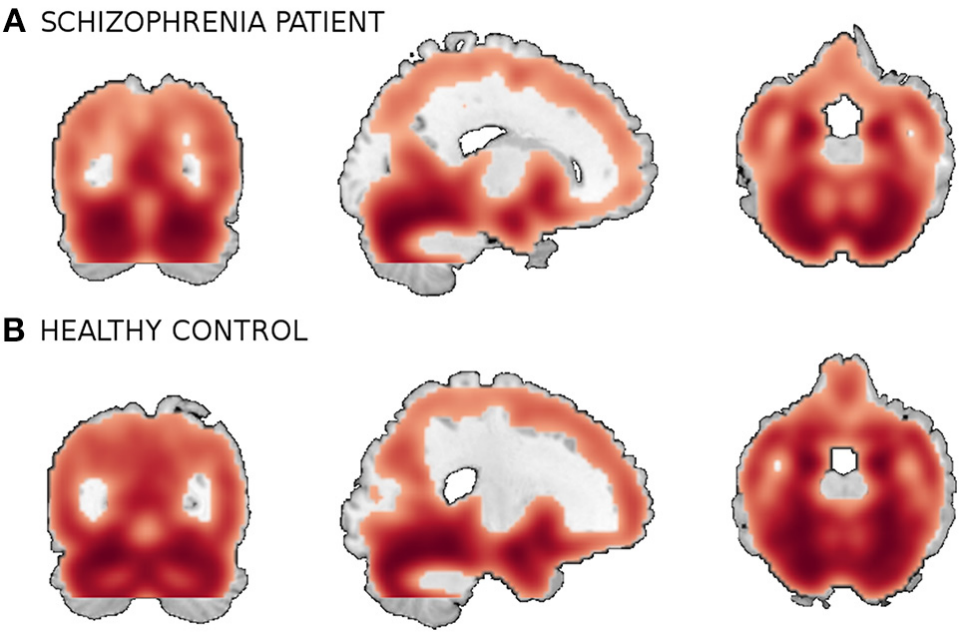
**A smoothed gray matter segmentation of a training sample of **(A)** a patient and **(B)** a healthy control**.

We use a combined data from four separate schizophrenia studies conducted at Johns Hopkins University (JHU), the Maryland Psychiatric Research Center (MPRC), the Institute of Psychiatry, London, UK (IOP), and the Western Psychiatric Institute and Clinic at the University of Pittsburgh (WPIC) (the data used in Meda et al., 2008). The combined sample comprised 198 schizophrenia patients and 191 matched healthy controls and contained both first episode and chronic patients (Meda et al., 2008). At all sites, whole brain MRIs were obtained on a 1.5T Signa GE scanner using identical parameters and software. Original structural MRI images were segmented in native space and the resulting gray and white matter images then spatially normalized to gray and white matter templates respectively to derive the optimized normalization parameters. These parameters were then applied to the whole brain structural images in native space prior to a new segmentation. The obtained 60465 voxel gray matter images were used in this study. Figure 5 shows example orthogonal slice views of the gray matter data samples of a patient and a healthy control.

The main question of this section is to evaluate the effect of the depth of a DBN on sMRI. To answer this question, we investigate if classification rates improve with the depth. For that we sequentially investigate DBNs of 3 depth. From RBM experiments we have learned that even with a larger number of hidden units (72, 128, and 512) RBM tends to only keep around 50 features driving the rest to zero. Classification rate and reconstruction error still slightly improves, however, when the number of hidden units increases. These observations affected our choice of 50 hidden units of the first two layers and 100 for the third. Each hidden unit is connected to all units in the previous layer which results in an all to all connectivity structure between the layers, which is a more common and conventional approach to constructing these models. Note, larger networks (up to double the number of units) lead to similar results. We pre-train each layer via an unsupervised RBM and discriminatively fine-tune models of depth 1 (50 hidden units in the top layer), 2 (50-50 hidden units in the first and the top layer respectively), and 3 (50-50-100 hidden units in the first, second and the top layer respectively) by adding a softmax layer on top of each of these models and training via the back propagation (see Figure 4). Table 1 summarizes parameter values used in the training.

**Table 1:** **Parameter settings for training RBMs at the pre-training and for the feed forward networks at the discriminative fine-tuning**.

**Depth**	**Pre-training**				**Fine tuning**			
	**Input**	**1**	**2**	**3**	**Input**	**1**	**2**	**3**
Dimension	60465	50	50	100	60465	50	50	100
Unit type	Gaussian	Logistic	Logistic	Logistic	–	Logistic	Logistic	Logistic
Dropout probability	0.2	0.5	0.5	0.5	0.7	0.5	0.5	0.75
*L*_1_ Regularization	–	0.1	0.01	0.001	–	0.001	–	–
Learning rate	–	0.01	0.01	0.001	–	0.01	0.1	1*e*–8

We estimate the accuracy of classification via 10-fold cross validation splitting the 389 subject dataset into 10 approximately class-balanced folds. At each step using 9 of the ten folds for pre-training and fine-tuning a DBN of a given depth, we use the same data to optimize parameters of the classifiers and only then perform evaluation on the left out fold. The process is repeated 10 times. We train the rbf-kernel SVM, logistic regression and a k-nearest neighbors (knn) classifier using activations of the top-most hidden layers in fine-tuned models to the training data of each fold as their input. The testing is performed likewise but on the test data. We also perform the same 10-fold cross validation on the raw data. Table 2 summarizes the precision and recall values in the F-scores and their standard deviations.

**Table 2:** **Classification on fine-tuned models (test data)**.

**Depth**	**Raw**	**1**	**2**	**3**
SVM F-score	0.68 ± 0.01	0.66 ± 0.09	0.62 ± 0.12	0.90 ± 0.14
LR.F-score	0.63 ± 0.09	0.65 ± 0.11	0.61 ± 0.12	0.91 ± 0.14
KNN F-score	0.61 ± 0.11	0.55 ± 0.15	0.58 ± 0.16	0.90 ± 0.16

**Figure 6:**
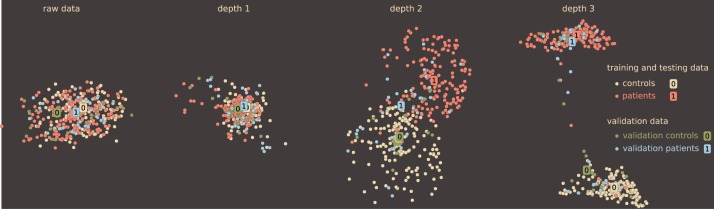
**Effect of a DBN's depth on neighborhood relations**. Each map is shown at the same iteration of the algorithm with the same *k* = 50. The color differentiates the classes (patients and controls) and the training (335 subjects) from validation (54 subjects) data. Although the data becomes separable at depth 1 and more so at depth 2, the DBN continues distilling details that pull the classes further apart.

All models demonstrate a similar trend when the accuracy only slightly increases from depth-1 to depth-2 DBN and then improves significantly. Table 2 supports the general claim of deep learning community about improvement of classification rate with the depth even for sMRI data. Improvement in classification even for the simple knn classifier indicates the character of the transformation that the DBN learns and applies to the data: it may be changing the data manifold to organize classes by neighborhoods. Ideally, to make general conclusion about this transformation we need to analyze several representative datasets. However, even working with the same data we can have a closer view of the depth effect using the method introduced in Section 3.2. Although it may seem that the DBN does not provide significant improvements in sMRI classification from depth-1 to depth-2 in this model, it keeps on learning potentially useful transformaions of the data. We can see that using our simple local neighborhood-based embedding. Figure 6 displays 2D maps of the raw data, as well as the depth 1, 2, and 3 activations (of a network trained on 335 subjects): the deeper networks place patients and control groups further apart. Additionally, Figure 6 displays the 54 subjects that the DBN was not train on. These hold out subjects are also getting increased separation with depth. This DBN's behavior is potentially useful for generalization, when larger and more diverse data become available.

Our new mapping method has two essential properties to facilitate the conclusion and provide confidence in the result: its already mentioned local properties and the deterministic nature of the algorithm. The latter leads to independence of the resulting maps from the starting point. The map only depends on the models parameter *k*—the size of the neighborhood—and the data.

### 3.4. A large-scale huntington disease data

In this section we focus on sMRI data collected from healthy controls and Huntington disease (HD) patients as part of the PREDICT-HD project (www.predict-hd.net). Huntington disease is a genetic neurodegenerative disease that results in degeneration of neurons in certain areas of the brain. The project is focused on identifying the earliest detectable changes in thinking skills, emotions and brain structure as a person begins the transition from health to being diagnosed with Huntington disease. We would like to know if deep learning methods can assist in answering that question.

For this study T1-weighted scans were collected at multiple sites (32 international sites), representing multiple field strengths (1.5T and 3.0T) and multiple manufactures (Siemens, Phillips, and GE). The 1.5T T1 weighted scans were an axial 3D volumetric spoiled-gradient echo series (≈1 × 1× 1.5 mm voxels), and the 3.0T T1 weighted scans were a 3D Volumetric MPRAGE series (≈ 1× 1 × 1 mm voxels).

The images were segmented in the native space and the normalized to a common template. After correlating the normalized gray matter segmentation with the template and eliminating poorly correlating scans we obtain a dataset of 3500 scans, where 2641 were from patients and 859 from healthy controls. *We are not studying the depth effect on performance in this section. Our goal with this imbalanced dataset is to evaluate if DBNs could facilitate discovery.* For that, we have used all of the 3500 scans in this imbalanced sample to pre-train and fine tune the same model architecture (50-50-100) as in Section 3.3. Here, however, we only used the complete depth 3 model.

**Figure 7:**
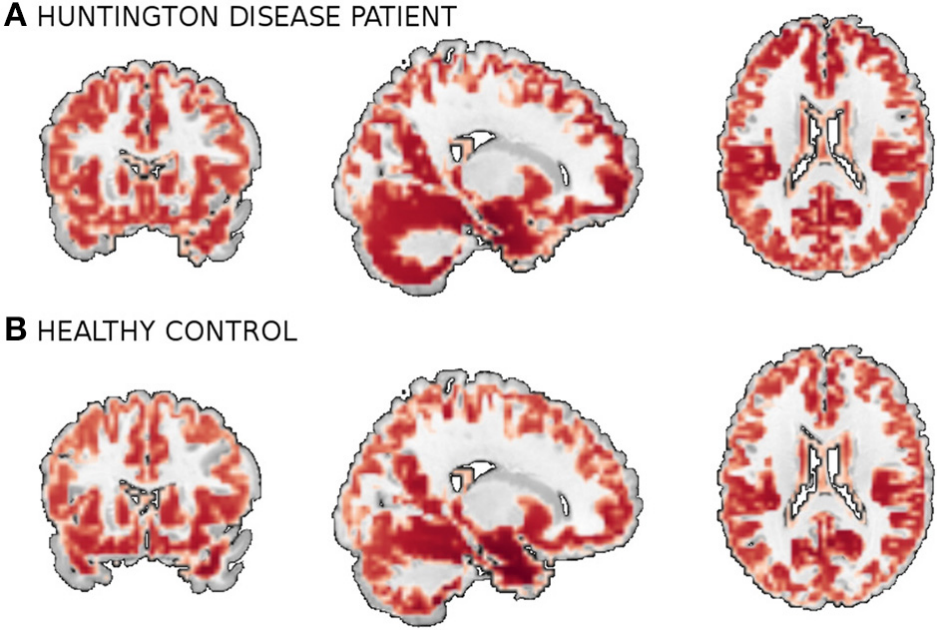
**A gray matter of MRI scans of an HD patient (A) and a healthy control (B)**.

**Figure 8:**
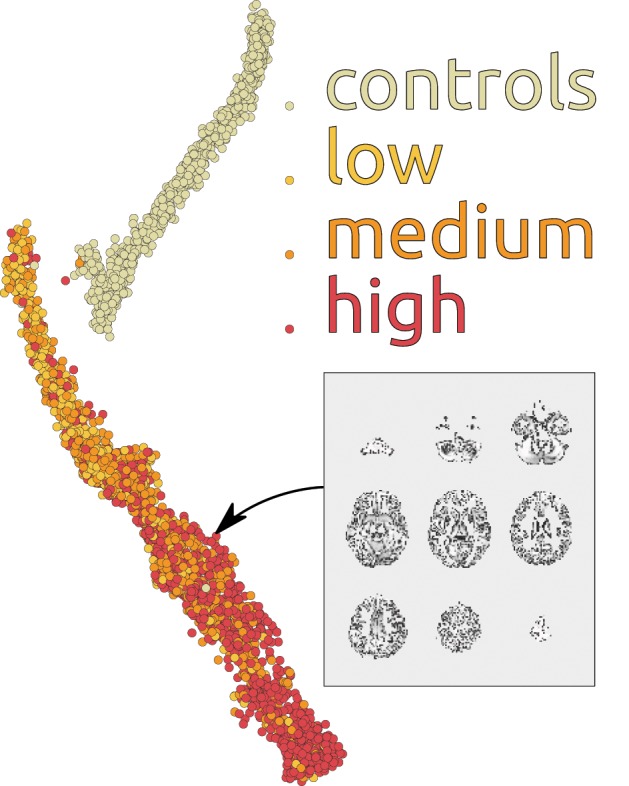
**Patients and controls group separation map with additional unsupervised spectral decomposition of sMRI scans by disease severity**. The map represents 3500 scans.

To further investigate utility of the deep learning approach for scientific discovery we again augment it with the embedding method of Section 3.2. Figure 8 shows the map of 3500 scans of HD patients and healthy controls build on using 100 dimensional representations learned by our depth 3 model. Each point on the map is an sMRI volume, shown in Figures 7, 8. Although we have used the complete data (all 3500 scans) to train the DBN, discriminative fine-tuning had access only to binary label: control or patient. In addition to that, we have information about severity of the disease from low to high. We have color coded this information in Figure 8 from bright yellow (low) through orange (medium) to red (high). The network^3^ discriminates the patients by disease severity which results in a spectrum on the map. Note, that neither t-SNE (not shown), nor our new embedding see the spectrum or even the patient groups in the raw data. This is a important property of the method that may help support its future use in discovery of new information about the disease.

## 4. Conclusions

Our investigations show that deep learning has a high potential in neuroimaging applications. Even the shallow RBM is already competitive with the model routinely used in the field: it produces physiologically meaningful features which are (desirably) highly focal and have time course cross correlations that connect them into meaningful functional groups (Section 5). The depth of the DBN does indeed help classification and increases group separation. This is apparent on two sMRI datasets collected under varying conditions, at multiple sites each, from different disease groups, and pre-processed differently. This is a strong evidence of DBNs robustness. Furthermore, our study shows a high potential of DBNs for exploratory analysis. As Figure 8 demonstrates, DBN in conjunction with our new mapping method can reveal hidden relations in data. We did find it difficult initially to find workable parameter regions, but we hope that other researchers won't have this difficulty starting from the baseline that we provide in this paper.

### Conflict of interest statement

The Associate Editor Dr. Poline declares that, despite having collaborated with author Dr. Jessica A. Turner, the review process was handled objectively. The authors declare that the research was conducted in the absence of any commercial or financial relationships that could be construed as a potential conflict of interest.

## Footnotes

^1^More detailed information regarding participant demographics is provided in Swanson et al. (2011).

^2^The task is described in more detail in Calhoun et al. (2008) and Swanson et al. (2011).

^3^Note, the embedding algorithm does not have access to any label information.
