# Supplementary material for: Deep learning for neuroimaging: a validation study
Source: Front Neurosci. 2014 Aug 20;8:229. doi: 10.3389/fnins.2014.00229 (PMC4138493; doi:10.3389/fnins.2014.00229)
Supplement: Supplementary file 1 [file DataSheet1.PDF]

SUPPLEMENTARY MATERIAL

Functional network connectivity (FNC) is a measure of interaction between intrinsic networks of the brain (**Allen et al.**, 2012). In our case this amounts to cross-correlations of subject specific time courses of each of the hidden unit expressed in a correlation matrix. The correlation matrices for both RBM and ICA results on the fMRI dataset of Section 2.3 are provided in Figure S1, where the ordering of components is performed separately for each method. Each network is named by their physiological function but we do not go in depth explaining these in the current paper. For RBM, modularity is more apparent, both visually and quantitatively. Modularity, as defined in (**Rubinov and Sporns**, 2011), averages 0.40 ± 0.060 across subjects for RBM, and 0.35 ± 0.056 for ICA. These values are significantly greater for RBM ($t = 7.15, p < 1e{-}6$ per the paired t-test). In experiments with synthetic data FNC (in part presented in Figure 2c), we found that RBM tends to overestimate strong cross-correlations. Note that the scale of correlation values for RBM and ICA is different in Figure S1, which highlights this observation.

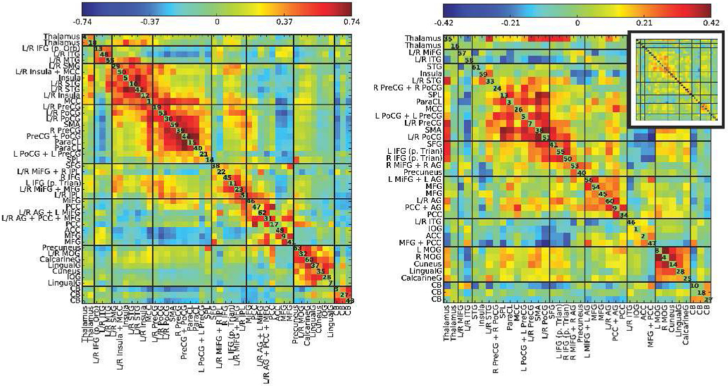

Figure S1: Correlation matrices determined from RBM (left) and ICA (right), averaged over subjects. Note that the color scales for RBM and ICA are different (RBM shows a larger range in correlations). The correlation matrix for ICA on the same scale as RBM is also provided as an inset (upper right). Feature groupings for RBM and ICA were determined separately using the FNC matrices and known anatomical and functional properties.

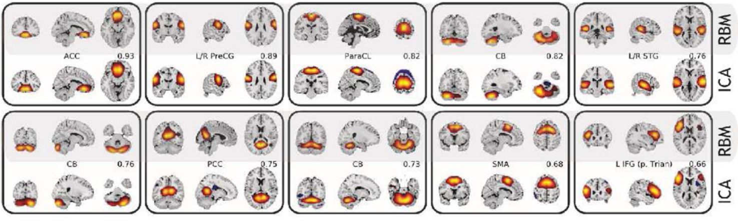

Figure S2: Sample pairs consisting of RBM (top) and ICA (bottom) SMs thresholded at 2 standard deviations. Pairing was done with the aid of spatial correlations, temporal properties, and visual inspection. Values indicate the spatial correlation between RBM and ICA SMs.
